# Long-term antidepressant use in general practice: a qualitative study of GPs’ views on discontinuation

**DOI:** 10.3399/BJGP.2020.0913

**Published:** 2021-04-20

**Authors:** Maria Donald, Riitta Partanen, Leah Sharman, Johanna Lynch, Genevieve A Dingle, Catherine Haslam, Mieke van Driel

**Affiliations:** Primary Care Clinical Unit, Faculty of Medicine, The University of Queensland, Brisbane, QLD, Australia.; Rural Clinical School, Faculty of Medicine, The University of Queensland, Hervey Bay, QLD, Australia.; School of Psychology, Faculty of Health and Behavioural Sciences, The University of Queensland, St Lucia, QLD, Australia.; Primary Care Clinical Unit, Faculty of Medicine, The University of Queensland, Brisbane, QLD, Australia.; School of Psychology, Faculty of Health and Behavioural Sciences, The University of Queensland, St Lucia, QLD, Australia.; School of Psychology, Faculty of Health and Behavioural Sciences, The University of Queensland, St Lucia, QLD, Australia.; Primary Care Clinical Unit, Faculty of Medicine, The University of Queensland, Brisbane, QLD, Australia.

**Keywords:** antidepressant, antidepressant discontinuation, depression, general practice, long-term antidepressant use, qualitative research

## Abstract

**Background:**

There is considerable concern about increasing antidepressant use, with Australians among the highest users in the world. Evidence suggests this is driven by patients on long-term use, rather than new prescriptions. Most antidepressant prescriptions are generated in general practice, and it is likely that attempts to discontinue are either not occurring or are proving unsuccessful.

**Aim:**

To explore GPs’ insights about long-term antidepressant prescribing and discontinuation.

**Design and setting:**

A qualitative interview study with Australian GPs.

**Method:**

Semi-structured interviews explored GPs’ discontinuation experiences, decision-making, perceived risks and benefits, and support for patients. Data were analysed using reflexive thematic analysis.

**Results:**

Three overarching themes were identified from interviews with 22 GPs. The first, ‘not a simple deprescribing decision’, spoke to the complex decision-making GPs undertake in determining whether a patient is ready to discontinue. The second, ‘a journey taken together’, captured a set of steps GPs take together with their patients to initiate and set-up adequate support before, during, and after discontinuation. The third, ‘supporting change in GPs’ prescribing practices’, described what GPs would like to see change to better support them and their patients to discontinue antidepressants.

**Conclusion:**

GPs see discontinuation of long-term antidepressant use as more than a simple deprescribing decision. It begins with considering a patient’s social and relational context, and is a journey involving careful preparation, tailored care, and regular review. These insights suggest interventions to redress long-term use will need to take these considerations into account and be placed in a wider discussion about the use of antidepressants.

## INTRODUCTION

Evidence suggests a steady rise in antidepressant use reported in the UK, Netherlands, the US, and Australia can be explained by an increase in long-term use.^1^^–^^6^ While antidepressants can be effective in major depression,^7^ meta-analytic evidence indicates they are no more effective than placebo in less severe depressive disorders.^8^^–^^10^ Indeed, an emerging body of research suggests considering treatment discontinuation for some long-term users.^11^^–^^14^ Prolonged use is a concern because of the potential for avoidable side effects and serious adverse events, including upper gastrointestinal bleeding, hyponatremia, stroke, falls and fractures,^15^^–^^17^ alongside emotional numbing, weight gain, sleep disturbance, sexual dysfunction,^18^^,^^19^ and loss of personal agency.^20^

General practice is the setting in which depression is most commonly treated and antidepressants are initiated and maintained.^2^^,^^21^^,^^22^ And while most long-term use is associated with recurrent depression, one third of long-term users are in remission, with no clear reason for continued use.^21^^,^^23^ This suggests considerable scope within general practice for treatment re-evaluation and discontinuation of antidepressants.

Effective change in prescribing practices must be built on an understanding of GPs’ and patients’ perspectives and the context in which it takes place. A 2010 UK study found a widespread belief among GPs and patients in the efficacy of antidepressants and only few concerns about side effects, resulting in little impetus for change.^24^ A more recent systematic review synthesised the patient perspective.^25^ Barriers include patient dependence, unpleasant withdrawal symptoms, and fear of relapse. Also, when patients hold the view that depression is a long-term condition, biological in nature, and are pessimistic about its curability, they are more reluctant to consider discontinuation.^25^

Key facilitators for patients include confidence in one’s ability to cope, a self-identity focused on being healthy or one’s true self, and a sense of stability in life circumstances, as well as guidance and support from significant others, including their prescribing doctor. Despite this central role in initiating discontinuation, GPs may continue to prescribe as they are reluctant to disturb the therapeutic alliance.^24^ This patient–GP dyad has been highlighted in other research.^26^ Nevertheless, in their review, Maund *et al*^25^ argue there are insufficient data to capture the GP perspective, a gap that the present paper, exploring GPs’ insights about long-term antidepressant use, addressed.

**Table:** How this fits in

Most antidepressant prescriptions are initiated and continued in general practice and discontinuation can be challenging. Understanding GPs’ insights into decision-making around discontinuing long-term antidepressant use is needed to underpin change. This study pointed to discontinuation as a journey for the GP–patient dyad that is not a simple deprescribing decision but is built on relationship and grounded in enabling social context.

## METHOD

### Setting, participants, and recruitment

A convenience sample of GPs was recruited through advertising in two Australian Primary Health Networks’ newsletters, emails to professional networks, and flyers distributed through university teaching networks and GP practices. Interested GPs contacted the research team via email, and further information describing the rationale for the research was provided via an information sheet. All GPs provided informed, written consent to participate and were provided the questions prior to interview allowing them the opportunity to consider their viewpoints.

### Data collection

Interviews were semi-structured and informed by published literature on long-term antidepressant prescribing^24^^,^^26^ and the clinical experience of the research group. The interview guide (Box 1) was piloted with two GPs (data not included in analysis but available from authors on request) and revised before study commencement. The interviews were conducted by telephone or face-to-face by four authors with previous interview experience, including two with training in psychology and two GP clinician-researchers. Interviews continued until data saturation occurred, with no new ideas identified in responses. Interviewers recorded field notes during and after interviews. All interviews were audiorecorded, transcribed verbatim, checked, and anonymised.

**Box 1.** Interview topic guide

**Experiences and opinions**
Can you think of a patient for whom discontinuing long-term antidepressant use was successful and describe the experience and the process used? (long-term >2 years) Now can you think of a patient for whom discontinuing long-term antidepressant use was unsuccessful and describe how the experience was different and why you think the attempt was unsuccessful? We know distinguishing between relapse and withdrawal symptoms can be challenging, can you talk a little about your experience with this in relation to discontinuing long-term antidepressant use? Can you describe your monitoring and reviewing process for patients on antidepressant therapy? In general terms, what do you think about the role of antidepressants in treating depression?
**Decision-making** What factors play a role in your decision to continue or discontinue long-term antidepressant use? What do you see as the risks of stopping long-term antidepressant treatment? What do you see as the benefits of stopping long-term antidepressant treatment?
**Support for GPs** To what extent do you feel you have sufficient knowledge and experience to advise patients correctly and to help them with discontinuing antidepressants? What would you need to discontinue long-term antidepressant use with more patients?
**Non-pharmacological support for patients** What non-pharmacological interventions do you refer patients to? Are you aware of any low-intensity non-pharmacological interventions (such as online CBT, guided self-help, wellbeing apps or websites) for patients and do you ever suggest them to your patients? The idea of social prescribing in general practice (or community referral to social groups) is gaining momentum as a model of care, do you have any thoughts about it and its use in supporting patients who are discontinuing long-term antidepressant use?

### Analysis

Data were analysed using reflexive thematic analysis, an approach appropriate for identifying patterns of meaning across data and suitable for questions related to understanding people’s experiences.^27^^,^^28^ Two members of the research team, independently read a selection of transcriptions, coding and adding notes as they familiarised themselves with the data.

Initial codes were created through collaboration of these two principal coders. They then continued to analyse the data by reading and re-reading all transcripts with regular review of the codes, combining, clustering, and collapsing them, ultimately generating prototype themes. Four further authors read and coded a pragmatically selected subset of transcripts. Team agreement was sought as the themes were reviewed, refined, and reordered ensuring a concise and meaningful representation of the data. NVivo (version 12) was used to aid data management and analysis.

## RESULTS

Twenty-two interviews were conducted between June and September 2019. Interviews lasted between 20 and 60 minutes (mean 35 minutes), 16 were by telephone and six face-to-face.

Participants were aged between 33 and 73 years (mean age 47 years), with 13 male and nine female, 16 practised in the state of Queensland (with 2, 3, and 1 located in New South Wales, Victoria, and South Australia, respectively), and the number of years since graduation ranged from 5 to 34 years. Practice postcodes indicated all GPs worked in either urban (*n* = 11) or inner-regional (*n* = 11) settings. A review of GP practice websites indicated six GPs had a specific interest in mental health.

Three main themes provide the overarching structure under which GPs’ insights are discussed within each subtheme. Box 2 lists these with a summary statement reflecting the focus of each subtheme.

### Theme 1. Discontinuation of long-term use of antidepressants is not a simple deprescribing decision

GPs emphasised discontinuing long-term antidepressant use was broader than a medical deprescribing decision. This theme describes the thorough exploration of the patient’s context, the complexities involved in shared decision-making, and extensive consideration required to assess preparedness for discontinuation.

**Box 2.** Themes and subthemes

**Theme 1: Discontinuation of long-term use of antidepressants is not a simple deprescribing decision**	
**Subtheme**	**Focus**
Assessing patient preparedness	Patients’ life circumstances are as important as recovery from depression in assessing patient preparedness for discontinuation. GPs acknowledged patient relationships with antidepressants that can disrupt preparedness to discontinue.
Subjective and relational decision-making	GPs described decision-making about discontinuation in intuitive and relational terms.
Weighing up benefits and risks	GPs recognised patient empowerment and sense of recovery as potent motivators for ceasing long-term use.
**Theme 2: Discontinuation of long-term use of antidepressants is a journey taken together by patient and GP**	
**Subtheme**	**Focus**
Planting the seed for change	GPs valued a process of careful preparation for discontinuation.
Co-designing a personalised plan	A tailored plan of action enables GPs and patients to increase the likelihood of successful discontinuation: a gradual dose reduction plan and proactive relapse plan are considered crucial.
Care continues during and after discontinuation	GPs emphasised regular review and encouragement of social and lifestyle supports during and beyond discontinuation.
**Theme 3: Supporting change in GPs’ prescribing practices**	
**Subtheme**	**Focus**
Redressing repeat prescribing as the quick fix	GPs expressed distrust in prescribing norms and felt a need to shift away from ‘set and forget’ attitudes.
Inadequate evidence to support discontinuation	Discussions with patients about discontinuation would be facilitated by better evidence about the harms of long-term use.
Practice-based change	GPs expressed well-communicated ideas about practice level change that would help them discontinue antidepressants.
Solutions beyond general practice	Discontinuation of long-term antidepressant use at the level of the GP–patient alliance will be leveraged by action at the broader system level (for example, social and policy).

#### Assessing patient preparedness

Personal and social circumstances were viewed as equally important as recovery from depression in assessing patient readiness. Having a stable relationship, employment, presence of social supports, low financial stress, awareness of triggers, engagement in self-care, and healthy lifestyle were repeatedly advocated as critical:

*‘And so it’s normally at a stage when they’re quite stable mentally and they feel pretty good in themselves, they haven’t really needed any other psychological interventions, and that’s usually when I find they’re more responsive to coming off therapy when there’s no other crises going on in their life.’*

(GP04, M[Male], 38 years [age])

Patient reluctance was a potent barrier to GPs broaching the topic of discontinuation. They used language to describe this reluctance such as *‘pretty adamant’* (GP20, F[female], 58), *‘once they’ve made up their mind’* (GP14, F, 33), and ‘ *hesitant’* (GP05, M, 41 years).

GPs acknowledged patient relationships with antidepressants may undermine their preparedness and noted patients need to want to stop. In particular, several GPs felt the relationship some patients have with their antidepressant is part of their identity and this may be a barrier:

*‘I think the single biggest factor is really the patient factor. If the patient really finds that they like the sedation, they’ve got a sense of “I can’t change … This is who I am …”’*

(GP10, F, 54 years)

Failed previous attempts can moderate patients’ future readiness, raising the level of concern among GPs about enabling unsuccessful attempts:

*‘Because if you stop them at the wrong time you lose the opportunity to stop them again in the future, sometimes.’*

(GP12, M, 36 years)

There were circumstances where GPs would not attempt discontinuation even if indicated. They mentioned the importance of respecting patients’ preference to remain on their medication. A few GPs indicated that for older patients who have been on antidepressants for a long-time (in some cases decades) *‘getting depressed again at seventy-five usually is not worth the risk.’* (GP09, M, 54 years) *.* Others suggested dose reduction rather than discontinuation was an adequate outcome in some circumstances, particularly when the patient is reluctant to cease, is in an unsafe or unstable environment, has inadequate social supports, or has experienced significant trauma:

*‘If a patient wasn’t willing. I mean, I’d have the conversation with them and I’d try to explain it. But, for example, this elderly lady that I’m thinking of … she just didn’t feel like she could manage without it. And I felt that what I understood of her childhood trauma and the fact that I was meeting her in her seventies, I had to just trust her judgement on that. And that maybe she did have quite profound ongoing impacts of that trauma that meant she needed to continue the medication or at least felt that she did. So I just had to respect her decision on it.’*

(GP07, F, 46 years)

#### Subjective and relational decision-making

GPs described their decision-making in intuitive and relational terms. They mentioned trust, being aware of the person’s situation and highlighted the subjective nature of the decision-making:

‘ *So, you feel like you really are doing this alone and there’s not really clear guidelines about when you should stop medication; it’s all a little bit subjective … So it is sort of based on intuition and a good relationship with the patient, so that is sometimes not as clear-cut as following a guideline and that might also be what stops us discontinuing it. It’s quite a subjective area.*’

(GP14, F, 33 years)

GPs repeatedly likened the process of antidepressant discontinuation to smoking cessation or dependence management, with one GP describing them as *‘modern day mother’s little helper’* and a *‘crutch’* (GP10, F, 54 years), while another said:

*‘But to me it’s a little bit like quitting smoking, they’ve got to be ready to want to stop, even if you tell them about the side effects and that they don’t need to be on it as many times as you want but if they’re not ready then in my experience it won’t go very well.’*

(GP14, F, 33 years)

The strength of the GP–patient alliance influences decision-making about whether to raise discontinuation, or challenge a patient’s reluctance to try or indeed try again. A weak therapeutic alliance is a barrier:

*‘Are they my regular patient … are they likely to come back to me? I don’t think that ... if there was you know the inability to follow-up easily that I would do it.’*

(GP22, F, 52 years)

#### Weighing up the benefits and risks

GPs agreed there are many benefits and few risks to ceasing long-term use when no longer indicated. The reversal or removal of side effects, the removal of emotional numbing, reduced medication burden, reduced polypharmacy risks, and removing the burden of cost are all motivators for discontinuation. Many GPs’ emphasised the empowering effect of being released from medication reliance. They felt discontinuing could increase a patient’s wellbeing by virtue of them being able to manage symptoms and recognising they were in control rather than feeling the medication was controlling the symptoms:

*‘The patient gains new life, they feel like, “I don’t need it! I’ve regained my life. I don’t need to be dependent on anything.”’*

(GP16, M, 62 years)

Few GPs expressed concern about the risk of suicide, the risk of relapse was the most common concern:

*‘The risks are that you can make a stable situation unstable and so that is a risk that you have to weigh up.’*

(GP22, F, 52 years)

### Theme 2. Discontinuation of long-term use of antidepressants is a journey taken together by the patient and the GP

GPs emphasised there is no standardised approach, it is about finding the appropriate strategy for each patient. Discontinuation was described as a journey taken together with ongoing discussions over time to review progress and better prepare patients to optimise outcomes. Making clear to patients they are not doing this alone is key and requires being fluid and responsive to the patient and their circumstances.

#### Planting the seed for change

Joint reflection with patients about why antidepressants were initiated and using the right language, can increase the likelihood of successful discontinuation. GPs used tentative language such as *‘broach the subject’* (GP14, F, 33 years), or said they *‘keep bringing it up.’* (GP19, M, 33 years) with the patient from time to time. They considered themselves to be planting the seed:

*‘So, I would say “can we consider getting you off this?” And either acting on the thought there if they’re agreeable or planting the seed that maybe next time we could do that or in a few months* ’ *time when their life’s treating them a bit better to give it a go.’*

(GP11, M, 38 years)

GPs argued for setting-up a period of preparation where enablers of success could be put in place:

*‘We plan the timing of the first step down, we make sure it’s a good time in their life ... not at a stressful time.’*

(GP20, F, 58 years)

Some GPs use standardised tools for identifying symptom change as part of the discussion with patients about discontinuation:

*‘I don’t want to spend too much time on tools … I find it’s a useful measurement to discuss with the patients about how effective the antidepression treatment is. So, if I can say — look your K10 when we started you on the antidepressants you were 35 and now you’re down to 22 … I think it’s having a good effect.’*

(GP10, F, 54 years)

#### Co-designing a personalised plan

Gradual tapering was mentioned almost universally as critical for discontinuation. Many GPs recognised that tapering plans need to be personalised as weaning periods are hard to establish due to variation in antidepressant type and dose, the message was clear — they go as slow as needed and generally slower than withdrawal regimens suggest:

*‘I just go slow as I think the patient* [needs] *… and look we might get to a dose that’s not therapeutic but it’s sort of a more just easing them back in to nothing and just showing them that they can do this without the medication.’*

(GP14, F, 33 years)

Being proactive about relapse planning is central to the process. Talking to patients about how they will recognise if they are not doing well, possible warning signs, and what they might do if they notice these signs, such as calling on social supports, returning to the GP, or re-engaging with mental health support were all mentioned as important:

*‘And I really like them to have, at least, talked about their other strategies they might use to manage things given that life does throw people curve balls. … Trying to, out loud, describe what they might do to both prevent and also monitor things … What are their early warning signs? … What might they do if they notice that? When would they come back to see me, when would they go and see their counsellor? Would they call on their support people? Just having a bit of a plan around that.*’

(GP20, F, 58 years)

GPs felt inadequate discontinuation planning meant patients may mistake withdrawal for relapse so preparing patients by helping them distinguish between withdrawal and relapse is key, as is preparing patients for the possibility that ceasing long-term use may be uncomfortable:

*‘I warn patients as much as possible about the fact that they will get some withdrawal … I’ll also suggest to them that it will be uncomfortable but it’s likely to be short-term ... I try to distinguish between the immediate sort of 2-week* [withdrawal] *effect rather than the 2-month* [relapse] *effect, and tell them what might feel different about those scenarios, and ... that one is quite highly expected and the other ... hopefully won’t happen.’*

(GP10, F, 54 years)

#### Care continues during and after discontinuation

Regular review during discontinuation enables symptom monitoring and reinforcing the importance of adhering to lifestyle measures, such as exercise, diet, sleep hygiene, social supports, and possibly psychological support. The value of frequent and regular review was stressed:

*‘But if they’ve been on it for a long period of time then I’ll want to see them every two weeks or even every week depending on how comfortable they feel. Because if they’re not supported at the cessation stage then they will most likely say I will need to be on this for the rest of my life.’*

(GP19, M, 33 years)

GPs had mixed opinions about whether a patient who was feeling ‘well’ would want to engage with psychological support. Some felt re-engaging with psychological support and social groups may make discontinuation more successful:

*‘I won’t say a better time but it might be at a time that if they’re better they’re actually more likely to go along* [to non-pharmacological support]. *Whereas, at the beginning stage it’s very hard … when people don’t have motivation to get out of bed ... So, maybe when they’re recovered it’s better.’*

(GP11, M, 38 years)

### Theme 3. Supporting change in GPs’ prescribing practices

This theme conveys GPs’ ideas about the individual, practice, and societal changes needed to support them to discontinue long-term antidepressants.

#### Redressing repeat prescribing as the quick fix

Providing a script for antidepressants was seen as an easy GP action that might allay symptoms as well as comfort patients. For example, one GP noted it was often easy to ‘ *let sleeping dogs lie*’ and ‘ *don’t fix it if it ain’t broke.’* (GP02, M, 56 years).

Prescribing and repeat prescribing were viewed in some cases as easier than attempting to address patients’ often complicated economic, social, and personal issues through lifestyle change and talking therapies:

*‘So, I suppose it is easier to prescribe a medication than it is to counsel, look at non-pharmacological things. It may feel like you’re doing more by giving a medication than just saying “oh, we’ll try these other things.”’*

(GP02, M, 56 years)

Some GPs saw antidepressants used in a way that ‘ *disempowered* ’ (GP10, F, 54 years) and caused ‘ *learned helplessness*’ (GP10, F, 54 years), and wanted to redress the approach to them as a panacea for all distress.

In particular, some GPs felt it was imperative to be clear about the limited duration of antidepressant use on their commencement, thereby avoiding any preestablished beliefs patients may have about antidepressants being for life, and priming them for the discontinuation conversation in future:

*‘So, when I do start someone on antidepressants I say “Well I don’t believe this should be something you take forever ... I intend to review it every 3 months and then to come off after you’ve been stable for at least 6 months.”’*

(GP17, M, 36 years)

While patients’ attitudes and reluctance to discontinue can reinforce and encourage the use of antidepressants as a quick fix *,* GPs also commented on the need to redress their own ‘ *set and forget’* (GP22, F, 52 years) attitude:

*‘There is a little bit of an underlying rule that once you start this medication nobody thinks to stop it.’*

(GP14, F, 33 years)

#### Inadequate evidence to support discontinuation

GPs distinguished between evidence about side effects and evidence about adverse health outcomes of long-term use. The former has a strong evidence-base well-articulated by GPs. Yet, there was an awareness that despite the widespread use of antidepressants, GPs mentioned they were not aware of research investigating potentially adverse health outcomes of long-term use.

GPs felt it was difficult to have the conversation with patients or justify to them why they should discontinue their use of antidepressants without being able to communicate the harms of long-term use:

*‘I think that long-term evidence is actually quite limited about any harms of long-term use. It is very difficult because most of the trials with antidepressants are short-term.’*

(GP09, M, 54 years)

#### Practice-based change

Most GPs felt they had sufficient knowledge and experience to provide adequate advice to patients around discontinuation. Yet, increased education for GPs in a variety of mediums, patient handouts, and better support for GPs were all mentioned as potentially helpful. Opportunities for auditing and benchmarking was a common recommendation:

*‘I think … clinical audits are good things … maybe not an in depth clinical audit but just something as part of a continuous professional development program.’*

(GP02, M, 56 years)

They spoke about removing well-known service delivery obstacles that interfere with their ability to deliver comprehensive and independent mental health care in general practice:

*‘I worry that it has something to do with the push to do 6 minute medicine and that this is the easier option sometimes than spending time with the patient and talking with the patient ... I hope we’re educating our GPs better than that and we’re empowering them to do more without medication.’*

(GP22, F, 52 years)

GPs pointed out there is conflicting advice about the rate at which antidepressants should be reduced. Some preferred to be guided by their own clinical experience and protocols. Current guidelines provide little clear advice for managing patients who are long-term users:

*‘So, in terms of guidelines, I’m not aware of any if there’s any.’*

(GP16, M, 62 years)

Several GPs mentioned the ongoing influence of the pharmaceutical industry in general practice and its potential contribution to medication overuse, including long-term use of antidepressants:

*‘And, just finally, I think that the biological model of management of depression and anxiety has been so strong for so long that drug companies are really at the centre and core of all of this and that unfortunately the majority of doctors are getting their education from drug companies directly or indirectly.’*

(GP10, F, 54 years)

#### Solutions beyond general practice

Having better access to affordable psychologists, including in-house psychologists and other non-pharmacological supports at the point of discontinuation were seen as enhancers of success:

*‘… we’ve got access to psychologists but it’s not ready access ...’*

(GP08, F, age unknown)

Although being interviewed about a clinical deprescribing process, GPs were repeatedly drawn to make non-medical observations about the use of antidepressants and many sought solutions within the broader social and policy environment.

Some believed there was a need to shift the conversation to one that considered how mental health and antidepressants were viewed in the community, and how current attitudes and beliefs could contribute to a culture of overprescribing and low rates of discontinuation:

*‘It’s probably nothing to do with doctors, it’s actually changing the conversation in our society about what causes anxiety and why … the management of anxiety baseline is not a medication, it’s not a drug, but it’s about finding ways to manage it by changing your lifestyle.’*

(GP10, F, 54 years)

## DISCUSSION

### Summary

Effective change in long-term antidepressant prescribing must be built on an understanding of GPs’ and patients’ perspectives, yet few studies have explored GPs’ perspectives. This study aimed to address this gap. The study revealed that discontinuation of long-term antidepressants is more than a simple deprescribing decision. It requires a thorough exploration of the patient’s social and personal circumstances, beliefs, and potential risks and benefits to assess preparedness. The GPs interviewed in this study described discontinuation as a journey taken together with the patient, involving ongoing discussions over time to review goals, and preparing patients by setting-up adequate support before, during, and after discontinuation to optimise outcomes.

GPs stressed the need for better support to undertake the complex and time-consuming work involved in ceasing long-term use and redressing the ‘set and forget’ attitude originating in the ease/convenience of prescribing and repeat prescribing, as well as gaining evidence of the harms of long-term use, and seeking solutions within broader social and policy environments.

### Strengths and limitations

The collection of in-depth data grounded in GPs’ experiences of discontinuing long-term antidepressant use with their patients was a strength of this study. Although some GPs interviewed had a specific interest in mental health, recruitment methods allowed for GPs with a range of skills to be included. Nevertheless, given the nature of the sampling method used, the study may not have fully captured the breadth of opinion and knowledge of GPs, especially those with limited experience in working with patients in this context or interest in being interviewed about it.

The findings reflect the views of GPs working in urban and regional contexts and may not reflect the experiences of rural GPs. They also capture an Australian GP’s perspective; though, the non-health-system related themes are likely relevant to GPs in other health systems. Multiple interviewers allowed flexibility in scheduling of interviews and use of a semi-structured interview schedule with regular interviewer meetings supports consistency in approach. However, this could be a strength as it may have added to the diversity of opinions captured. Likewise, data analysis was undertaken not only by GPs, but also by those with a background in mental health providing broader perspectives.

### Comparison with existing literature

Most studies have focused on the patient perspective of long-term antidepressant use and suggest a key barrier to discontinuation is a patient’s belief that their GP is responsible for initiating discussions about discontinuation.^25^ While this underlines the need for GP vigilance in raising the idea of discontinuation with patients, the findings highlight the complex decision-making GPs go through in deciding whether to raise the issue. The few previous studies investigating GPs’ perspectives show that patients’ difficult life circumstances are a barrier to GPs initiating discontinuation.^25^ The extent to which GPs in this study considered life circumstances in assessing patient preparedness for discontinuation reinforces this and highlights the social and relational nature of shared decision-making in this context. This aligns this study’s findings with those of Wentink *et al,*^29^ who call for a comprehensive Decision Aid to facilitate more confident shared decision-making, and emphasising, as others have, that respect for individual agency, an appreciation of context, and a respectful empathic approach to practice, are essential to delivering patient-centred care.^30^

This study’s findings further align with Wentnick *et al* in that discontinuation was expressed by GPs as a journey or ‘ *process of discontinuation’ ^29^*, highlighting the steps or ‘ *practicalities’ ^29^* of discontinuation including planting the seed, a tailored plan of action with gradual dose reduction and proactive relapse planning, and ensuring care continues during and after discontinuation. Gradual personalised tapering was mentioned almost universally as important for discontinuation supporting previous research suggesting an approach where GPs tailor care to individual needs, may be better than a standardised schedule.^29^ Several GPs interviewed in this study stressed the need to warn patients about the possibility of unpleasant withdrawal reactions when stopping antidepressants.

A 2015 review by Davies and Read found withdrawal symptoms are quite common, can be severe, and often last from weeks to several months.^31^ While there is ongoing discussion about the incidence and severity of antidepressant withdrawal effects, there is increasing acknowledgement that withdrawal symptoms are variable with calls to update treatment guidelines and for prescribers to advise patients about the possibility of withdrawal reactions.^31^^,^^32^

The emphasis GPs in this study placed on patients regaining a sense of empowerment and control, reinforces research showing patient’s self-identity as ‘healthy’ or regaining their ‘true-self’ can facilitate discontinuation.^24^ Despite this acknowledgement, repeat prescribing of antidepressants was seen as an easy GP action contributing to ‘set and forget’ attitudes and concomitant overprescribing. Previous research showing lack of time for GP review as an important barrier to discontinuation,^24^^,^^26^^,^^33^ is consistent with these findings and emphasises GPs need the clinical time for complex contextual and relational decision-making around discontinuing antidepressants. Although the GPs interviewed in this study recognised that, ultimately a culture shift within and beyond the profession is necessary if attitudes to antidepressant use are to change.

### Implications for practice and research

The implications for GPs and patients arising from this research are numerous and contribute to a small but growing body of research that can inform safe and effective ways to support discontinuing long-term antidepressant use in general practice.^24^ Stopping unnecessary long-term use needs to start at initiation, and GP education and training must give adequate attention to prescribing, repeat prescribing, and deprescribing. A request for a repeat prescription should never be considered a ‘just a script’ consultation, it should always come with a review of whether this medication is still required. Resetting the ‘set and forget’ attitude for both GPs and patients is critical and could be assisted by prescribing audits to facilitate review of antidepressant use.

The findings confirm the commitment in primary care to assessment of the whole person^34^ and support a generalist (not simply psychiatric) decision-making process at the time of initial diagnosis and prescription.^35^ The findings suggest that GPs should be recognised, remunerated, and offered clinical time to do their complex contextual and relational decision-making around diagnosis, prescribing, and deprescribing of antidepressants. Future research could build on this work, to design and test multi-modal interventions that assist GPs and the GP–patient dyad to navigate the multi-layered journey of antidepressant discontinuation. Aligning assessment and treatment of depression with generalist approaches to the person could build GP confidence in the complex relational and contextual decision-making skills that underpin the collaborative process of discontinuation of antidepressants.
